# Securing Biomechanical Data Quality: A Comprehensive Evaluation of On-Board Accelerometers for Shock and Vibration Analysis

**DOI:** 10.3390/s25154569

**Published:** 2025-07-23

**Authors:** Corentin Bosio, Christophe Sauret, Patricia Thoreux, Delphine Chadefaux

**Affiliations:** 1 Arts et Métiers Institute of Technology, EPF Engineering School, Université Sorbonne Paris Nord, IBHGC–Institut de Biomécanique Humaine Georges Charpak, F-75013 Paris, France; corentin.bosio1@univ-paris13.fr (C.B.); christophe.sauret@invalides.fr (C.S.); patricia.thoreux@aphp.fr (P.T.); 2 Centre d’Etudes et de Recherche sur l’Appareillage des Handicapés, Institution Nationale des Invalides, F-75007 Paris, France; 3 Hôpital Hôtel-Dieu, AP-HP, F-75004 Paris, France

**Keywords:** accelerometer, metrology, vibration, shock

## Abstract

**Highlights:**

**What are the main findings?**

- **Blue Trident** demonstrates the highest accuracy in shock amplitude and timing (relative errors < 6%), while **Xsens** provided stable measurements under low-frequency vibrations.
- **Shimmer 3** showed significant signal variability, raising concerns about its reliability for high-precision biomechanical analysis.

**What is the implication of the main finding?**

- **Blue Trident** is a strong candidate for applications requiring precise shock measurement, such as impact analysis in biomechanics.
- Proper sensor selection is essential to ensure data quality in biomechanical applications involving dynamic movements.

**Abstract:**

(1) On-board accelerometers are increasingly employed in real-world biomechanics to monitor vibrations and shocks. This study assesses the accuracy, repeatability, and variability of three commercially available inertial measurement units (IMUs)—Xsens, Blue Trident, and Shimmer 3—in measuring vibration and shock parameters relevant to human motion analysis. (2) A controlled laboratory setup utilizing an electrodynamic shaker was employed to generate sine waves at varying frequencies and amplitudes, as well as shock profiles with defined peak accelerations and durations. (3) The results showed that Blue Trident demonstrated the highest accuracy in shock amplitude and timing, with relative errors below 6%, while Xsens provided stable measurements for low-frequency vibrations. In contrast, Shimmer 3 exhibited considerable variability in signal quality. (4) These findings offer critical insights into sensor selection based on specific application needs, ensuring optimal accuracy and reliability in dynamic measurement environments. This study lays the groundwork for improved IMU application in biomechanical research and practical deployments. Future research should continue to investigate sensor performance, particularly in angular motion contexts, to further enhance motion analysis capabilities.

## 1. Introduction

Humans frequently encounter vibrations and shocks in diverse contexts such as vehicular transport, industrial machinery, and athletic performance [1]. These dynamic exposures may induce discomfort [2,3], reduced performance, or contribute to chronic musculoskeletal pathologies [4,5]. Understanding how vibrations and shocks propagate through the human body is essential to improving ergonomic design, preventing injuries, and enhancing safety protocols. Despite technological advances, accurately measuring these phenomena remains challenging due to the complexity of human biomechanics and the non-linear behavior of the musculoskeletal system, which responds differently depending on factors such as load, direction, and frequency of movement.

Vibrations involve sustained oscillatory motion, while shocks are sudden, high-magnitude events [1]. Although both are measured in terms of acceleration, their temporal and energetic properties differ significantly. Consequently, sensor selection must consider key attributes such as the sampling rate (how frequently data are recorded, in Hz), dynamic range (maximum and minimum measurable acceleration), and temporal resolution (precision in capturing rapid changes over time) to ensure fidelity in capturing these phenomena. Addressing these metrological challenges is a key aspect of accurate data acquisition. To address these distinct requirements, it is essential to consider sensor parameters like measurement range, response time, weight, ease of integration, wireless synchronization, and the type of attachment (e.g., taped, strapped, or embedded), which can significantly affect signal quality. These collectively determine a sensor’s fitness for field deployment and its reliability in capturing dynamic motion.

In occupational settings, where individuals are often exposed to dynamic loads despite static postures, Griffin [1] identifies amplitude and frequency as the core parameters that define and influence vibration exposure. Whole-body vibrations associated with effects on health, activities, and comfort range from 0.5 to 100 Hz, with acceleration magnitudes between 0.01 and 10 m/s^2^ [1,6,7]. Meanwhile, shock accelerations can reach levels of 61 to 129 m/s^2^, particularly in high-impact environments such as high-speed boats [8]. In sports biomechanics, understanding the dynamic environments experienced by individuals requires precise measurement parameters, particularly when investigating short-duration impacts. Bartlett [9] identified the acquisition frequencies necessary for accurately studying such activities. For instance, tennis requires a minimum acquisition frequency of 50 Hz for general motion analysis [9]. The frequency content transmitted to the upper limb does not exceed 150 Hz, meaning an acquisition frequency (Fs) of 500 Hz is generally sufficient. However, due to the brief nature of impacts, a higher sampling rate (e.g., 500–1000 Hz) may be needed to extract key shock characteristics, such as amplitude [9]. In tennis, when placing an accelerometer on the racket, the frequency content can reach 1500 Hz, which, according to Nyquist’s theorem, requires a minimum sampling frequency (Fs) of 3000 Hz to ensure adequate resolution [10].

For seated individuals exposed to whole-body vibration (WBV), research indicates that the relevant frequency range falls between 4 and 100 Hz, with movement amplitudes ranging from 0.01 to 10 m/s^2^ [1,8,11,12,13,14]. During walking, the frequency ranges from 4 to 8 Hz, with tibial acceleration amplitudes below 2.4 g and impact forces between 1.7 g and 3.3 g, depending on footwear [15]. For running, ground reaction forces typically generate tibial accelerations between 3 and 8 Hz, with linear acceleration amplitudes below 10 g [16]. These accelerations can lead to maximal impact forces, reaching 14.5 ± 5.8 g at the foot and decreasing at the distal tibia to 11.5 ± 5.1 g [16]. Studies also report impacts between 7 and 10 g depending on the speed and surface [17]. Jump landings, especially from moderate heights, typically induce vertical accelerations of 8–12 g at the tibia and up to 15 g at the heel [9].

In upper limb activities, sports like tennis and golf produce much higher localized accelerations. For example, during tennis, racket impacts can reach 163 ± 8 g for a forehand, with 26.02 ± 11.8 g at the wrist and 7.38 ± 2.35 g at the elbow [9,10,18,19]. Similarly, in golf, swing frequencies near 100 Hz result in 150 ± 31 g at the club head and over 5000 g upon ball impact [9,20,21].

In the field of biomechanics, inertial measurement units (IMUs) have become a widely utilized technology for capturing kinematic data. This growing use has led to a sharp rise in research publications on the topic, especially in the last decade [22]. Compared to traditional wired accelerometers, IMUs offer key advantages such as wireless operation, compact design, on-board data logging, and ease of deployment in real-world conditions, making them particularly suitable for biomechanical applications. While valid piezoelectric and piezoresistive sensors exist, they are not readily available yet in a compact, lightweight, and easily on-boarded format due to the required conditioning.

In this context, comparative assessment of IMU accelerometer accuracy, repeatability, and signal quality under shock and vibration conditions would be of great interest to inform users of the actual capabilities and limitations of current IMU-based accelerometers. For that purpose, a primary challenge is the selection of appropriate sensors capable of accurately capturing dynamic phenomena, such as vibration and shock, both of which are quantified through acceleration despite their distinct characteristics.

Among the many IMUs available, this study focuses on Xsens, Blue Trident, and Shimmer 3—three models commonly used in human motion analysis. Xsens (Xsens Technologies, Enschede, The Netherlands) has undergone extensive validation across various activities, demonstrating a high correlation with the motion analysis gold standard, a camera-based motion capture, in specific planes. It has been extensively used for gait analysis (ankle, knee, and hip flexion/extension at 100 Hz), as well as running and tennis (whole-body motion, excluding the hand, in the sagittal plane at 240 Hz). Notably, over 500 studies have utilized Xsens for joint kinematic assessments [23,24]. Blue Trident (Dual-g IMU, Oxford, UK, low-g ±16 g sampling rate 1125 Hz, high-g ±200 g sampling rate 1600 Hz) has been validated for running metrics like step count and impact load, with more than 15 studies supporting its use [25]. While Shimmer 3 (Wireless IMU, Shimmer, Ireland, D, ±2 to 16 g, [2–1024] Hz) lacks full validation, it has nonetheless been used in multiple peer-reviewed studies [26,27]. Despite strong interest and validation efforts for kinematic and specific dynamic measurements, broader dynamic validation, for vibration and shock settings, remains unexplored. This study addresses this gap by evaluating these three IMU models using standardized lab protocols designed to emulate biomechanically relevant scenarios.

After defining a specific methodology, this paper aimed to evaluate Xsens, Blue Trident, and Shimmer 3 on-board accelerometers in terms of precision, accuracy, and errors from shock and vibration. From the results of this evaluation, recommendations on the advantageous use of these different sensors are provided, as well as the use cases when the different sensors should be avoided.

## 2. Materials and Methods

### 2.1. Sensor Installation

Three commercially available on-board accelerometers were selected for this study, based on their widespread dissemination into the biomechanics community. Their characteristics are detailed in Table 1. Moreover, the commercial IMUs used in this study have uniform accelerometer specifications across the X, Y, and Z axes. Therefore, our analysis was simplified to a single axis, with the assumption that it reliably represents performance across all axes. While Xsens and Blue Trident do not disclose the specific accelerometers they integrate, the Shimmer 3 incorporates either the Kionix-KXRB35 (Kionix, Inc., Ithaca, NY, USA) or the STMicro LSM303DLHC (STMicroelectronics NV, Geneva, Switzerland), depending on the selected measurement range. To isolate intrinsic sensor performance, measurements were conducted in controlled conditions rather than real-world biomechanics, avoiding confounding factors such as placement variability or soft tissue artifacts.

**Figure 1:**
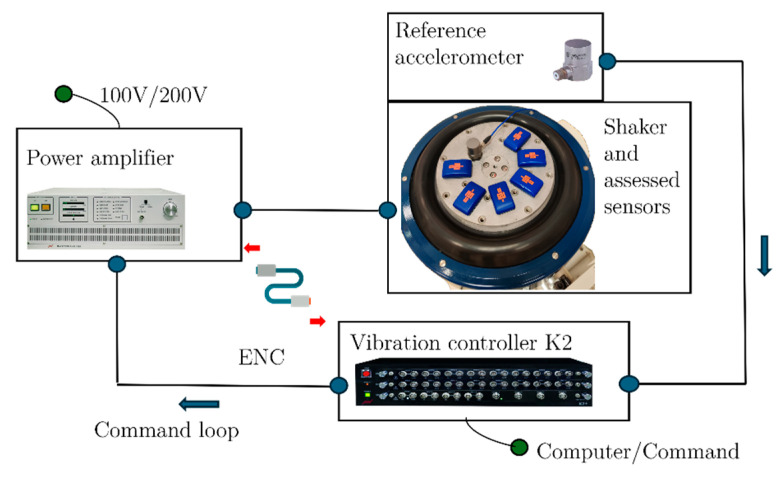
Closed-loop command of the electrodynamic shaker, studied and reference sensor.

The experiment used a closed-loop electrodynamic shaker (Figure 1) (IMV compact shaker series m-120, Osaka, JPN) to generate vertical signals with frequencies of [2–2000] Hz and amplitudes of [0–40] g. The closed loop was maintained by a control sensor (PCB Piezotronics 333C33, ICP, Buffalo, NY, USA, 100 mV/g, ±50 g pk, [0.5–10,000] Hz). The reference sensor used (PCB Piezotronics 333C33, ICP, Buffalo, NY, USA) was a calibrated, wired, piezoelectric accelerometer with a bandwidth of 0.5–10,000 Hz and a resolution of 5 × 10^−3^ m/s^2^ RMS, making it a suitable gold standard for laboratory-based shock and vibration measurements. The data acquisition was performed at a sampling frequency of 51,200 Hz. The closed-loop configuration, combined with the high-fidelity reference sensor, ensured precise signal generation and repeatability across trials.

The test signals—including sine waves and impact profiles—were controlled via a computer using a K2 interface (vibration controller and compatible software). All test sensors were rigidly mounted to the shaker platform using strong double-sided adhesive tape to guarantee consistent coupling and prevent motion artifacts. While the Shimmer sensor has a higher mass than the other models tested, its secure attachment and the stability of the shaker’s motion minimized the likelihood that mass influenced the results. Although the specific shaker model used here has not been referenced in prior validation studies, its performance under closed-loop control and the use of a calibrated reference sensor make it a robust and repeatable platform for comparative sensor evaluation (Figure 1).

### 2.2. Setpoint Signals

**Figure 2:**
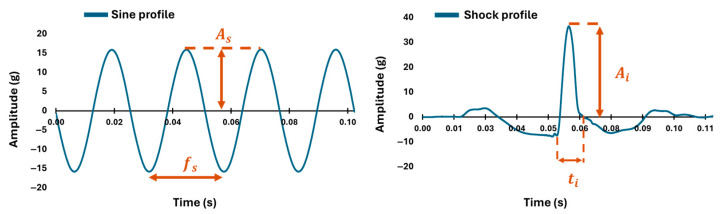
Tested signals and quantities of interest ($A_{s},\;f_{s},\;A_{i},\;t_{i}$, respectively, represent sine amplitude, sine frequency, impact amplitude, and shock duration) for sines and shocks.

Sensor performance was evaluated using two signal types: sinusoidal waves and shock pulses. Sine waves (Figure 2) were generated at frequencies of 2, 3, 40, and 100 Hz with amplitudes of 0.1, 0.25, 1, 16, and 35 g (4 conditions in total, for 20 trials). Shocks (Figure 2) were applied as double-sided half-sine waveforms, with peak accelerations of [10, 12, 17, 19, 24, 27, 31, 34, 38] g for the Blue Tridents and [5, 6, 9, 11, 13, 15, 17, 19, 21] g for the Xsens and Shimmer 3 sensors, lasting 7 ms and 13 ms, for every sensor (10 conditions in total, for 30 trials). These conditions were selected to emulate realistic biomechanical stimuli within experimental constraints. The chosen amplitudes spanned the usable measurement range of each device. While the Blue Trident’s full 200 g capacity was not tested due to hardware limitations, it was evaluated up to 38 g. The Xsens and Shimmer3 were tested across their full ±16 g range. These test conditions were selected to reflect the most meaningful operational range for each device, based on their sensor specifications and platform constraints.

The analysis focused on four key aspects: repeatability (consistency of results from repeated measurements under the same conditions), reproducibility (consistency of results across different sessions or setups), and data accuracy relative to the reference sensor. Metrics were computed separately for sine waves and shocks, based on processed raw data.

### 2.3. Processing Pipelines

To thoroughly evaluate sensor performance, data from the experimental protocol were processed. Repeatability was assessed through five successive measurements within the same experimental session [28]. Reproducibility was evaluated by conducting identical tests across five sessions, i.e., the same mounted disposition (Figure 1), on different days [28]. Intra-sensor variability was assessed by analyzing the standard deviation across five trials for each sensor model (Table 2, left section). Inter-sensor variability was assessed by computing the standard deviation of the trial means across different sensor technologies (6 Xsens, 5 Blue Tridents, and 3 Shimmer3). To validate measurement precision, the wireless sensors were benchmarked against the reference PCB Piezotronics 333C33 sensor (Table 2, middle section). Computed repeatability metrics, including mean values, standard deviation, and relative error, quantified deviations from the reference (Table 2, right section), providing a standardized measure of accuracy and reliability.

For each sine signal trial, the main frequency was estimated using a Fast Fourier Transform (FFT) applied to a 20 s window of the signal. Prior to the transformation, a Hanning window was applied to the time-domain data to minimize spectral leakage and improve frequency resolution. No additional filtering (such as low-pass or high-pass filtering) was applied during data analysis. The amplitude was determined by calculating the mean of the maximum peaks within a 20 s trial, which included at least 80 acceleration peaks per sine wave signal. The mean and standard deviation of the amplitudes across the five trials were then obtained, providing a measure of variability under coupled frequency and amplitude conditions.

The relative error was calculated for each trial by comparing the measured values of frequency and amplitude to those of the reference sensor (PCB), expressed as a percentage difference. These errors were summarized by calculating their mean and standard deviation, capturing deviations between the tested sensors and the reference sensor, as well as variability between different sensor units. Key metrics included the following:

- The percentage of relative error for frequencies and amplitudes, with precision maintained within ±5% of the nominal value, is often regarded as reliable data, in line with the tolerances specified by manufacturers (PCB Piezotronics’ ±5% tolerance for their accelerometers).
- The variability in both frequency and amplitude (inter- and intra-variability).

Furthermore, the signal-to-noise ratio (SNR) was computed to assess the quality of the signal relative to background noise. To compute the SNR, a high-resolution (HR) method was used. This method assumes that the gathered signals can be modeled as a noisy sinusoidal function:

$$x\left(k\right)=\sum_{i=1}^{d}s_{i}\cdot e^{jk\omega i}+n\left(k\right)$$

where $\omega_{i}\in (-\pi ,\pi )$ and $s_{i}$ are the normalized frequency and complex amplitudes of the i-th sinusoid, and n(k) is additive random noise. In this study, the signal consists of a single sinusoid at the dominant frequency, with the rest of the signal considered as noise. The SNR computation was further carried out by using the ESPRIT algorithm [29], enabling the separation of sinusoidal signals and noise. The mean and standard deviation of the SNR across the five trials were computed to provide additional insights into signal quality.

### 2.4. Shock Signals Processing and Quantities of Interest

For shock signals, two primary metrics were extracted from the data: the shock duration and the maximum amplitude peak. Shock duration was defined as the time interval between the moment the motion first exceeds a specified fraction of the maximum value (initial positive slope) and the moment it intersects the x-axis (Figure 2) [9]. The maximum amplitude peak represented the highest acceleration value recorded during the shock (Figure 2) and was directly obtained from the temporal data. For both metrics, the percentage of relative error was calculated with respect to the control sensor. Variability across trials and sensors was assessed by computing the standard deviation of the measured values, which provided insights into the following values of interest:

- The percentage of relative error for both the maximum amplitude peak and shock duration with respect to the data of the reference sensor.
- The variability in both amplitudes and shock duration (inter- and intra-variability).

All detailed numerical results, including trial-wise performance metrics across conditions and sensors, are presented in Table A1, Table A2, Table A3, Table A4, Table A5, Table A6, Table A7, Table A8 and Table A9 in Appendix A.

## 3. Results

**Figure 3:**
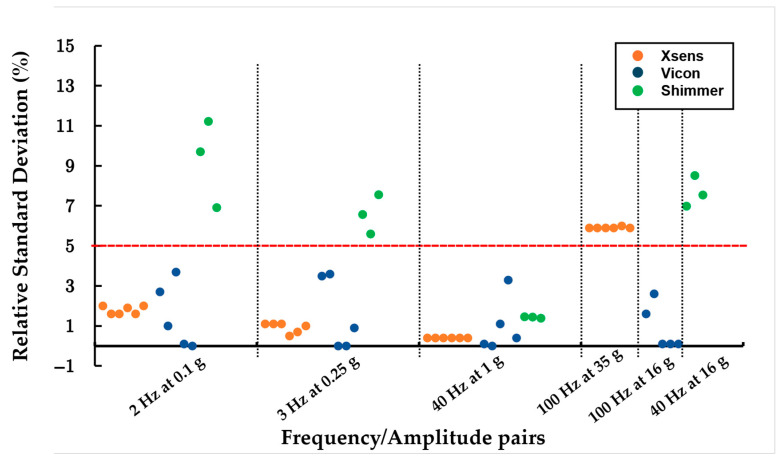
Amplitude standard deviation normalized to the input amplitude for the same trials and the same sensors: 6 Xsens, 5 Blue Tridents, 3 Shimmer3 (specifications in Table 1), considering frequency/amplitude pairs.

For the sine tests, most sensors demonstrated consistent repeatability across the five trials, with low normalized standard deviations (under 5%) observed for all frequency/amplitude pairs (Figure 3). However, Shimmer sensors exhibited significantly reduced repeatability, with normalized standard deviations exceeding 7% in several instances and amplitude relative errors reaching over 40%. Amplitude variations frequently exceeded 5%, especially for Vicon and Shimmer sensors, while frequency deviations were predominantly under 3% (Table A1, Table A2 and Table A3). The Vicon and Shimmer sensors showed some fluctuation in standard deviation percentages, yet values remained largely concentrated at low levels. In contrast, the Xsens sensors maintained stable standard deviations across all trials, following a consistent trend. The signal-to-noise ratio (SNR) repeatability varied among sensors and trials. The Xsens and Vicon sensors generally displayed low standard deviations, except in the high-frequency/amplitude trial, where the Xsens sensors showed a spread of over 60% around the mean, and the Vicon sensors reached around 9% (Table A1: Xsens sensor-to-sensor results, for frequency/amplitude pairs. Amplitude and frequency relative error and standard deviation. SNR mean and standard deviation results.).

The Shimmer sensors exhibited particularly low SNR values at 3 Hz, 0.25 g, with relative deviations exceeding 50%, whereas other trials showed more stable SNR distributions.

For the sine tests, amplitude intra-variability was notably high for Vicon and Shimmer sensors at lower-frequency/amplitude pairs but decreased as frequency and amplitude increased (Table A7). In contrast, Xsens sensors maintained minimal intra-variability across all conditions, demonstrating stable performance at higher frequencies. Frequency intra-variability was negligible for Xsens and Vicon sensors, whereas Shimmer sensors exhibited increased variability at specific pairs (40 Hz at 1 g) but remained stable otherwise. Signal-to-noise ratio (SNR) intra-variability was consistently high across all sensors, with Vicon and Shimmer sensors showing the greatest fluctuation (Table A9).

For shock signals, amplitude intra-variability remained low across all trials and sensors, with a normalized standard deviation below 3%. Shock timing intra-variability stayed within 10%, as acquisition frequencies were consistent across sensors of the same type. Notably, Xsens sensors demonstrated perfect synchronization, resulting in 0% timing variability across trials (Table A4, Table A5 and Table A6).

**Figure 4:**
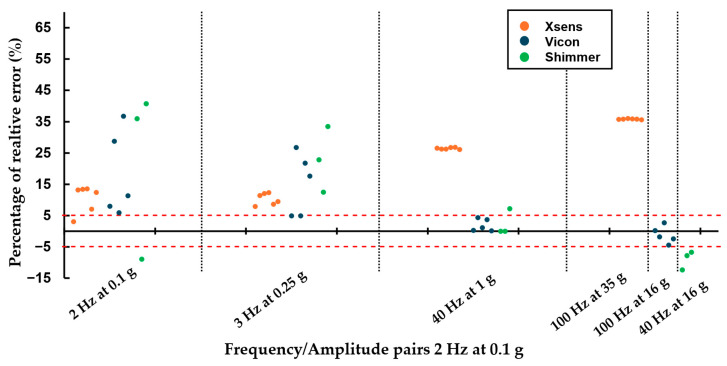
Amplitude percentage of error of each sensor relative to the reference sensor, sine pairs [Xsens, Blue Trident, Shimmer3].

For the sine tests, all sensors exhibited high inter-variability at low-frequency/amplitude trials (Figure 4). At higher-frequency/amplitude pairs, Vicon and Shimmer sensors showed reduced inter-variability, whereas Xsens sensors exhibited a distinct but consistent measurement pattern. Frequency inter-variability remained low, with all sensors measuring with high precision (lower than 5%), except for a notable spike in variability for Shimmer sensors at the 40 Hz and 1 g combination (Table A8). SNR intra-variability was high, almost over 5 (Table A9), across all trials for all sensors without any visible trends.

**Figure 5:**
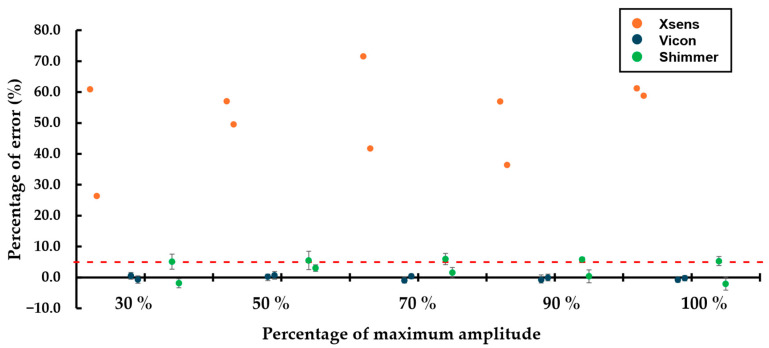
Percentage of relative error, for 16 g of maximum amplitude for the Xsens and Shimmer3 sensors and 35 g of maximum amplitude for the Blue Trident sensors, with 7 ms and 13 ms of shock time tests, sorted accordingly. The red dashed line indicates a 5% relative error threshold, commonly used as a benchmark for acceptable measurement accuracy.

For shock amplitude and timing measurements, Xsens sensors displayed significantly higher variability compared to Vicon and Shimmer sensors, which exhibited only slight differences (Figure 5). Variability between Xsens and Shimmer sensors was pronounced, while Vicon sensors showed a wider range of amplitudes, making a direct comparison less meaningful. However, this increased variability in Xsens sensors may be partially attributed to their lower sampling frequency, which limits their ability to accurately capture fast transient events such as shocks.

For the sine tests, accuracy varied across sensors. In terms of amplitude, Vicon and Shimmer sensors exhibited noticeable relative errors during low-frequency, low-amplitude trials, whereas Xsens sensors consistently maintained errors below 12% (Figure 4). At higher-frequency/amplitude trials, this trend reversed: Xsens sensors displayed errors exceeding 25%, while Vicon sensors remained around 7%, and Shimmer sensors stayed below 12%. Regarding frequency, all sensors maintained relative errors below 2%, indicative of high accuracy across this domain. For frequency measurements, all sensors maintained relative errors under 2%, indicating high accuracy in this domain. Signal-to-noise ratio (SNR) performance was generally favorable for Xsens sensors, consistently exceeding 20 across trials, except in high-frequency/amplitude conditions, where deviations increased. Shimmer sensors showed inconsistent performance across all trials, while Vicon sensors performed well at high-frequency/amplitude pairs (an SNR over 50) but underperformed at lower frequencies, reaching a mean SNR of 15 at 2 Hz (Table A1, Table A2 and Table A3).

For shock (Table A4, Table A5 and Table A6), in terms of amplitude, Vicon sensors recorded low and consistent relative errors across all trials, while Shimmer sensors had slightly higher but stable errors. Xsens sensors, however, exhibited noticeably higher relative errors throughout the trials. In terms of shock duration, relative errors varied significantly between sensors. Vicon sensors consistently reported low errors below 6%, whereas Xsens sensors recorded errors ranging from 55% to 75% for 13 ms shocks and 100% to 125% for 7 ms shocks. Shimmer sensors exhibited errors between 27% and 45% for 7 ms shocks but stayed below 14% for 13 ms shocks. These variations align with the sensors’ sampling frequency specifications.

## 4. Discussion

The present study systematically evaluated the performance of three commercially available IMUs—Xsens, Vicon, and Shimmer 3—under controlled vibration and shock conditions, with an emphasis on their suitability for biomechanical applications. Vicon sensors demonstrated the highest accuracy overall, consistently producing relative errors below 5% for both vibration and shock signals. This level of precision supports its use in dynamic environments requiring accurate peak measurements, such as sports involving high-impact ground contact or equipment strikes.

Xsens exhibited the best performance under low-frequency, low-amplitude conditions, showing stable behavior across vibration trials. Its performance profile makes it well suited for lower-intensity activities such as gait assessment, where tibial accelerations typically range from 1.7 to 3.3 g depending on gait phase and footwear [15,17]. This aligns with Xsens’s historical validation in clinical gait settings and controlled environments [24,30]. However, its lower sampling frequency limits its effectiveness for capturing fast transients, such as those encountered in sports impacts or jump landings, where tibial accelerations can reach between 8 and 12 g [9] and temporal resolution is critical for capturing brief shock durations [12].

Vicon, by contrast, captured these high-intensity signals with high fidelity. Specifically, it demonstrated relative errors of less than 1% for shock amplitude and below 6% for shock timing, outperforming the other sensors in these metrics. Its performance is particularly relevant for tasks like running and jumping, where foot strike can produce forces up to 14.5 ± 5.8 g at the foot and 11.5 ± 5.1 g at the tibia [16], or in equipment-based sports such as tennis and golf. In tennis, accelerations of up to 163 ± 8 g have been observed at the racket, with around 26 g reaching the wrist and approximately 7 g at the elbow [10,18,19]. These values fall well within Vicon’s dynamic range and sampling capabilities, which are necessary to avoid signal clipping and timing distortion [25]. Shimmer 3, while more variable in its outputs, still performed reasonably well in longer-duration shocks and may be appropriate for exploratory studies or applications where absolute accuracy is less critical [26].

Importantly, the performance differences observed across sensors have practical implications for real-world deployment. For instance, walking, occupational tasks, and seated posture analysis typically involve lower accelerations and can tolerate slightly higher measurement errors, favoring the use of Xsens or even Shimmer 3 in cost- or setup-sensitive contexts. In contrast, running and jumping require both sufficient dynamic range and high sampling frequency, capabilities most reliably met by Vicon. In activities involving brief and high-magnitude impacts, such as a golf swing or a tennis stroke, only sensors like Vicon—with sampling rates exceeding 1000 Hz and a dynamic range up to ±200 g—can ensure that key features like impact timing and peak amplitude are captured without distortion [20,21].

Table 3 reflects these distinctions, offering a task-specific framework for selecting IMUs based on both the physical demands of the activity and the technical limitations observed in this study. Rather than assuming functional equivalence across IMUs, these results demonstrate the need to match sensor capabilities to task-specific demands. Researchers and practitioners should weigh the sampling rate, dynamic range, and precision requirements against deployment constraints, including cost, size, and integration features. While Vicon offers broad versatility and superior accuracy, Xsens remains an excellent candidate for low-motion applications. Shimmer 3, though less precise, may be sufficient in monitoring tasks that prioritize accessibility and setup flexibility over data resolution.

## 5. Limitations

This study was confined to sensors already available in the laboratory, which may have excluded higher-performing or more recent technological advancements. While it provides empirical performance benchmarks, it does not define fixed thresholds for acceptable relative error or repeatability. Establishing such standards remains an important step for guiding sensor selection across applications and should be addressed in future research.

The assessment of sensor performance also revealed challenges in capturing high-amplitude, short-duration shocks, emphasizing the necessity of higher-frequency sampling systems to enhance measurement accuracy. Additionally, the analysis was limited to a single axis, potentially overlooking valuable multi-directional insights that could be crucial for real-world applications. Although the Vicon supports measurements up to 200 g, testing was limited to the maximum acceleration achievable within our laboratory constraints (38 g), thus not fully exploiting the sensor’s 200 g capacity. Finally, while this study focused on accelerometer performance, future research should also address the validation of gyroscope data—particularly angular velocity accuracy—as this remains increasingly relevant in both research and applied contexts. Prior work by Delgado-García [31], for example, reported gyroscope errors below 5% during tennis strokes, underscoring the potential for high-precision angular motion tracking.

## 6. Conclusions

This study provided a comparative evaluation of three widely used IMUs—Xsens, Blue Trident, and Shimmer 3—under controlled vibration and shock conditions. Using Fourier transforms and high-resolution signal processing techniques, we assessed their performance in terms of accuracy, repeatability, and signal quality. The findings underscore the critical importance of carefully considering sensor specifications with the demands of the intended biomechanical application, rather than assuming that all devices offer equivalent performance across contexts.

Blue Trident consistently demonstrated the highest accuracy and lowest variability, especially in shock amplitude and timing measurements, making it the most reliable for high-impact, high-frequency applications. Xsens showed strong performance for low-frequency, low-amplitude vibrations, particularly relevant to gait and posture studies, though its limited sampling rate reduced shock resolution. Shimmer 3, while less consistent in accuracy, particularly under low-energy vibration, still performed adequately in certain shock conditions, especially at higher durations, and may be useful in cost-sensitive or less precision-critical scenarios. These findings emphasize that sensor performance is not interchangeable and should be matched to the biomechanical demands of the target application. For example, Blue Trident is preferable in high-impact sports analysis, while Xsens suits subtle motion detection in clinical gait settings. Shimmer may be appropriate in experimental setups where ultra-high fidelity is not essential.

Future work could also build upon these insights by exploring the influence of various calibration techniques and the integration of advanced signal processing methods to further enhance sensor precision. Additionally, assessing sensor performance in real-world conditions, such as sports biomechanics and workplace ergonomics, may offer valuable perspectives on their practical applicability and potential optimizations.

Ultimately, this study advances the understanding of accelerometer performance in vibration and shock measurement, providing key insights into their capabilities and limitations. These findings serve as a foundation for optimizing sensor selection in diverse applications, ensuring both accuracy and reliability in dynamic measurement environments.

## Figures and Tables

**Table 1:** Key manufacturer specifications for the accelerometers in the selected IMUs.

Sensor Name	IMU (Name)	Accelerometer Model	Sampling Frequency (Hz)	Acceleration Range (g)	Axes	Mass (g)	Dimension (mm)
Xsens	Xsens-MTw Awinda	Unknown	120	±8, ±10, ±16, ±20	3-axis	16	47 × 30 × 13
Vicon	Vicon-Blue Trident	Unknown	Low-g 1125 and high-g 1600	Low-g ±16 and high-g ±200	3-axis	9.5	42 × 27 × 11
Shimmer	Shimmer-Shimmer3 IMU	Kionix-KXRB35 or STMicro LSM303DLHC	$2^{n}$ max 1024	±2, ±4, ±8, ±16	3-axis	30	65 × 32 × 12

**Table 2:** Amplitude mean, standard deviation, intra-sensor variability, and accuracy for Blue Tridents in repeated 40 Hz, 1 g trials.

Blue-tridents sensors	**Trials**					**Computed Data**			**PCB Data**
	trial 1	trial 2	trial 3	trial 4	trial 5	**Mean sensors**	**Standard deviation**	**Relative error %**	**PCB mean value**
Sensor 01306	1.003	1.004	1.003	1.002	1.000	**1.002**	**0.0016**	**0.20**	1.00
Sensor 02105	1.045	1.044	1.044	1.043	1.042	**1.044**	**0.0012**	**4.40**	1.00
Sensor 02113	1.014	1.014	1.012	1.011	1.009	**1.012**	**0.0021**	**1.20**	1.00
Sensor 03141	1.037	1.038	1.037	1.036	1.037	**1.037**	**0.0006**	**3.70**	1.00
Sensor 03142	1.001	1.001	1.001	1.000	1.001	**1.001**	**0.0004**	**0.10**	1.00
**Intra-variability (std)**	**0.0198**	**0.0197**	**0.0199**	**0.0199**	**0.0203**				
**Mean intra-variability**	**0.0199**								

**Table 3:** Sensor to activity for their specific values of interest.

Activity	Frequency	Movement Amplitude	Shock
Wheelchair	Xsens, Vicon, Shimmer	Xsens, Shimmer	Vicon, Shimmer
Walking	Xsens, Vicon, Shimmer	Xsens, Shimmer	Vicon, Shimmer
Running	Xsens, Vicon, Shimmer	Vicon, Shimmer	Vicon
Tennis	Xsens, Vicon, Shimmer	Vicon, Shimmer	-
Golf	Vicon, Shimmer	Vicon	-

## Data Availability

Data are contained within the article.

## Abbreviations

The following abbreviations are used in this manuscript:

IMU	Inertial Measurement Unit
WBV	Whole-Body Vibration
SNR	Signal-to-Noise Ratio

## Appendix A

**Table A1:** Xsens sensor-to-sensor results, for frequency/amplitude pairs. Amplitude and frequency relative error and standard deviation. SNR mean and standard deviation results.

Sensors	Frequency/Amplitude	Amplitude Relative Error (%)	Amplitude Relative Std	Frequency Relative Error (0.1%)	Frequency Std	Mean SNR	SNR Std
1	2 Hz at 0.1 g	3.1	0.020	−23.5	0	20	0.62
	3 Hz at 0.25 g	7.9	0.011	−7.2	0	26	0.64
	40 Hz at 1 g	26.5	0.004	−0.2	0	59	0.17
	40 Hz at 16 g	35.7	0.059	1	0	55	5.29
2	2 Hz at 0.1 g	13.2	0.016	−23.5	0	31	1.18
	3 Hz at 0.25 g	11.4	0.011	−7.2	0	36	1.22
	40 Hz at 1 g	26.3	0.004	−0.2	0	76	1.66
	40 Hz at 16 g	35.8	0.059	1	0	45	24.98
3	2 Hz at 0.1 g	13.4	0.016	−23.5	0	26	0.77
	3 Hz at 0.25 g	12.1	0.011	−7.2	0	32	0.34
	40 Hz at 1 g	26.3	0.004	−0.2	0	65	0.53
	40 Hz at 16 g	36.1	0.059	1	0	45	24.47
4	2 Hz at 0.1 g	13.5	0.019	−23.5	0	26	0.53
	3 Hz at 0.25 g	12.4	0.005	−7.2	0	35	0.49
	40 Hz at 1 g	26.7	0.004	−0.2	0	67	0.71
	40 Hz at 16 g	35.9	0.059	1	0	48	30.80
5	2 Hz at 0.1 g	7.1	0.016	−23.5	0	25	0.66
	3 Hz at 0.25 g	8.6	0.007	−7.2	0	32	0.36
	40 Hz at 1 g	26.8	0.004	−0.2	0	64	0.24
	40 Hz at 16 g	35.8	0.060	1	0	48	30.80
6	2 Hz at 0.1 g	12.4	0.020	−23.5	0	30	1.07
	3 Hz at 0.25 g	9.5	0.010	−7.2	0	30	0.29
	40 Hz at 1 g	26.2	0.004	−0.2	0	69	0.70
	40 Hz at 16 g	35.6	0.059	1	0	45	25.67

**Table A2:** Vicon sensor-to-sensor results, for frequency/amplitude pairs. Amplitude and frequency relative error and standard deviation. SNR mean and standard deviation results.

Sensors	Frequency/Amplitude	Amplitude Relative Error (%)	Amplitude Relative Std	Frequency Relative Error (0.1%)	Frequency Std	Mean SNR	SNR Std
1	2 Hz at 0.1 g	8.0	0.027	4.9	0.02	20	0.62
	3 Hz at 0.25 g	4.9	0.035	−12.9	0.02	26	0.64
	40 Hz at 1 g	0.3	0.001	0.7	0.03	59	0.17
	100 Hz at 35 g	0.2	0.016	0.0	0.00	55	5.29
2	2 Hz at 0.1 g	28.7	0.010	−4.9	0.02	16	0.51
	3 Hz at 0.25 g	26.8	0.036	−16.1	0.00	20	0.32
	40 Hz at 1 g	4.4	0.000	0.7	0.03	53	0.12
	100 Hz at 35 g	−1.8	0.026	0.0	0.00	59	5.12
3	2 Hz at 0.1 g	5.9	0.037	4.9	0.02	20	0.55
	3 Hz at 0.25 g	4.9	0.000	−12.1	0.02	29	0.56
	40 Hz at 1 g	1.1	0.011	0.7	0.03	58	0.27
	100 Hz at 35 g	2.7	0.001	0.0	0.00	45	2.89
4	2 Hz at 0.1 g	36.8	0.001	−4.9	0.02	8	2.35
	3 Hz at 0.25 g	21.8	0.000	−9.7	0.03	20	0.61
	40 Hz at 1 g	3.7	0.033	0.7	0.03	49	0.10
	100 Hz at 35 g	−4.4	0.001	0.0	0.00	51	3.07
5	2 Hz at 0.1 g	11.3	0.000	−4.9	0.02	21	0.82
	3 Hz at 0.25 g	17.7	0.009	−16.1	0.00	22	0.59
	40 Hz at 1 g	0.1	0.004	0.7	0.03	58	0.14
	100 Hz at 35 g	−2.4	0.001	0.0	0.00	54	4.37

**Table A3:** Shimmer sensor-to-sensor results, for frequency/amplitude pairs. Amplitude and frequency relative error and standard deviation. SNR mean and standard deviation results.

Sensors	Frequency/Amplitude	Amplitude Relative Error (%)	Amplitude Relative Std	Frequency Relative Error (%)	Frequency Std	Mean SNR	SNR Std
1	2 Hz at 0.1 g	35.9	0.097	−0.39	0.00	-	-
	3 Hz at 0.25 g	22.8	0.066	1.74	0.03	2	1.17
	40 Hz at 1 g	0.0	0.015	1.75	0.03	32	6.88
	100 Hz at 16 g	−12.4	0.070	0.11	0.00	22	0.14
2	2 Hz at 0.1 g	−9.0	0.112	−0.39	0.00	-	-
	3 Hz at 0.25 g	12.5	0.056	2.08	0.00	22	1.05
	40 Hz at 1 g	0.0	0.015	1.03	0.38	35	1.91
	100 Hz at 16 g	−7.8	0.085	0.11	0.00	25	0.06
3	2 Hz at 0.1 g	40.7	0.069	−0.39	0.00	-	-
	3 Hz at 0.25 g	33.5	0.076	2.08	0.00	5	3.33
	40 Hz at 1 g	7.2	0.014	1.72	0.00	32	0.09
	100 Hz at 16 g	−6.7	0.075	0.11	0.00	25	0.04

**Table A4:** Xsens sensor-to-sensor results, for shock signals. Amplitude and time of shock relative error and variability amongst sensors.

Time Command (s)	Amplitude (g)	Mean Amplitude (g)	Std Amplitude (g)	% Relative Error	Real Time Shock (s)	Sensor Shock (ms)	% Relative Error	Std Time (s)
7	5.6	2.2	0.004	60.90	8.87	20	125.6	0
7	9.4	4.0	0.020	57.07	9.47	20	111.1	0
7	13.3	3.8	0.013	71.59	9.77	20	104.8	0
7	17.2	7.4	0.038	56.94	9.96	20	100.8	0
7	18.9	7.3	0.050	61.22	9.82	20	103.6	0
13	6.2	4.5	0.023	26.40	18.85	30	59.2	0
13	10.9	5.5	0.022	49.57	19.26	30	55.8	0
13	15.0	8.7	0.019	41.77	17.09	30	75.5	0
13	19.2	12.2	0.035	36.44	17.21	30	74.3	0
13	21.0	8.7	0.040	58.79	18.50	30	62.2	0

**Table A5:** Vicon sensor-to-sensor results, for shock signals. Amplitude and time of shock relative error and variability amongst sensors.

Time Command (s)	Amplitude (g)	Mean Amplitude (g)	Std Amplitude (g)	% Relative Error	Real Time Shock (ms)	Sensor Shock (ms)	% Relative Error	Std Time (ms)
7	10.5	10.6	0.107	0.59	8.87	8.38	−5.6	0.71
7	17.4	17.5	0.141	0.26	9.47	9.50	0.3	0.68
7	24.5	24.2	0.265	−0.99	9.77	9.50	−2.7	0.68
7	31.3	31.1	0.475	−0.74	9.96	9.63	−3.4	0.68
7	34.7	34.5	0.344	−0.75	9.82	8.63	−12.2	0.68
13	11.7	11.7	0.139	−0.65	18.85	18.38	−2.5	0.34
13	19.4	19.6	0.248	0.67	19.26	18.38	−4.6	0.34
13	26.9	27.0	0.211	0.38	17.09	18.00	5.3	0.28
13	34.7	34.6	0.376	−0.04	17.21	17.75	3.2	0.56
13	38.5	38.4	0.324	−0.24	18.50	17.38	−6.1	0.52

**Table A6:** Shimmer sensor-to-sensor results, for shock signals. Amplitude and time of shock relative error and variability amongst sensors.

Time Command (s)	Amplitude (g)	Mean Amplitude (g)	Std Amplitude (g)	% Relative Error	Real Time Shock (s)	Sensor Shock (ms)	% Relative Error	Std Time
7	5.6	5.3	0.129	5.12	8.87	12.70	43.2	0.797
7	9.4	8.9	0.266	5.50	9.47	13.43	41.8	0.488
7	13.3	12.5	0.231	5.96	9.77	12.93	32.4	0.488
7	17.2	16.2	0.130	5.78	9.96	12.70	27.5	0.797
7	18.9	17.9	0.264	5.30	9.82	13.42	36.6	0.488
13	6.2	6.3	0.092	−1.87	18.85	20.75	10.1	0.488
13	10.9	10.6	0.109	3.05	19.26	21.00	9.0	0.563
13	15.0	14.8	0.247	1.58	17.09	19.53	14.3	0
13	19.2	19.1	0.398	0.38	17.21	18.80	9.3	0.935
13	21.0	21.4	0.452	−2.05	18.50	19.04	2.9	0.564

**Table A7:** Xsens, Vicon, and Shimmer sensors’ amplitude intra-variability relative percentage (orange) and inter-variability relative percentage (green).

		2 Hz at 0.1 g			3 Hz at 0.25 g			40 Hz at 1 g			100 Hz at 15 and 35 g		
		Xsens	Vicon	Shimmer	Xsens	Vicon	Shimmer	Xsens	Vicon	Shimmer	Xsens	Vicon	Shimmer
Excitation of 2 Hz at 0.1 g	Xsens	3.975	24.003	20.210									
	Vicon		12.582	19.174									
	Shimmer			23.666									
Excitation of 3 Hz at 0.25 g	Xsens				1.797	18.462	20.017						
	Vicon					9.333	9.826						
	Shimmer						9.205						
Excitation of 40 Hz at 1 g	Xsens							0.597	16.631	17.623			
	Vicon								1.779	3.274			
	Shimmer									4.129			
Excitation of 100 Hz at 15 and 35 g	Xsens										0.141	X	10.838
	Vicon											2.419	X
	Shimmer												2.088

**Table A8:** Xsens, Vicon, and Shimmer sensors’ frequency intra-variability percentage (orange) and inter-variability percentage (green).

		2 Hz at 0.1 g			3 Hz at 0.25 g			40 Hz at 1 g			100 Hz at 15 and 35 g		
		Xsens	Vicon	Shimmer	Xsens	Vicon	Shimmer	Xsens	Vicon	Shimmer	Xsens	Vicon	Shimmer
Excitation of 2 Hz at 0.1 g	Xsens	0.000	0.667	0.462									
	Vicon		0.453	0.860									
	Shimmer			0.000									
Excitation of 3 Hz at 0.25 g	Xsens				0.000	1.244	2.920						
	Vicon					0.612	3.341						
	Shimmer						0.000						
Excitation of 40 Hz at 1 g	Xsens							0.000	2.852	36.441			
	Vicon								0.078	36.696			
	Shimmer									12.990			
Excitation of 40 and 100 Hz at 15 and 35 g	Xsens										0.000	X	X
	Vicon											0.044	6.918
	Shimmer												0.000

**Table A9:** Xsens, Vicon, and Shimmer sensors’ SNR intra-variability (orange) and inter-variability (green).

		2 Hz at 0.1 g			3 Hz at 0.25 g			40 Hz at 1 g			100 Hz at 15 and 35 g		
		Xsens	Vicon	Shimmer	Xsens	Vicon	Shimmer	Xsens	Vicon	Shimmer	Xsens	Vicon	Shimmer
Excitation of 2 Hz at 0.1 g	Xsens	2.135	6.543	X									
	Vicon		4.796	X									
	Shimmer			X									
Excitation of 3 Hz at 0.25 g	Xsens				1.953	5.771	14.233						
	Vicon					3.379	10.428						
	Shimmer						8.240						
Excitation of 40 Hz at 1 g	Xsens							3.760	7.899	18.969			
	Vicon								3.848	12.617			
	Shimmer									1.674			
Excitation of 40 and 100 Hz at 15 and 35 g	Xsens										1.298	4.968	10.897
	Vicon											4.557	15.318
	Shimmer												1.216

## References

[1] Griffin M.J. (1990). Handbook of Human Vibration.

[2] Duarte M.L.M., De Araújo P.A., Horta F.C., Vecchio S.D., De Carvalho L.A.P. (2018). Correlation between Weighted Acceleration, Vibration Dose Value and Exposure Time on Whole Body Vibration Comfort Levels Evaluation. Saf. Sci..

[3] Maeda S., Futatsuka M., Yonesaki J., Ikeda M. (2003). Relationship between Questionnaire Survey Results of Vibration Complaints of Wheelchair Users and Vibration Transmissibility of Manual Wheelchair. Environ. Health Prev. Med..

[4] Dupuis H., Zerlett G. (1987). Whole-Body Vibration and Disorders of the Spine. Int. Arch. Occup. Environ. Health.

[5] Pope M.H., Magnusson M., Broman N.H., Hassont T. (1998). The Dynamic Response of Human Subjects while Seated in Car Seats. Iowa Orthop. J..

[6] Rasmussen G. (1983). Human Body Vibration Exposure and Its Measurement. J. Acoust. Soc. Am..

[7] (1997). Mechanical Vibration and Shock—Evaluation of Human Exposure to Whole-Body Vibration.

[8] Garme K., Burström L., Kuttenkeuler J. (2011). Measures of Vibration Exposure for a High-Speed Craft Crew. Proc. Inst. Mech. Eng. Part M J. Eng. Marit. Environ..

[9] Bartlett R. (2014). Introduction to Sports Biomechanics.

[10] Chadefaux D., Rao G., Androuet P., Berton E., Vigouroux L. (2016). Active Tuning of Stroke-Induced Vibrations by Tennis Players. J. Sports Sci..

[11] Kwarciak A.M. (2008). Curb Descent Testing of Suspension Manual Wheelchairs. J. Rehabil. Res. Dev..

[12] Hood S., McBain T., Portas M., Spears I. (2012). Measurement in Sports Biomechanics. Meas. Control.

[13] Lariviere O., Chadefaux D., Sauret C., Thoreux P. (2021). Vibration Transmission during Manual Wheelchair Propulsion: A Systematic Review. Vibration.

[14] Waga T., Ura S., Nagamori M., Uchiyama H., Shionoya A. (2020). Influence of Material on Wheelchair Vibrations. Proceedings.

[15] Lafortune M.A. (1991). Three-Dimensional Acceleration of the Tibia during Walking and Running. J. Biomech..

[16] Chadefaux D., Gueguen N., Thouze A., Rao G. (2019). 3D Propagation of the Shock-Induced Vibrations through the Whole Lower-Limb during Running. J. Biomech..

[17] James K.A., Corrigan P., Lanois C., Huang C.-H., Davis I.S., Stefanik J.J. (2023). Association of Tibial Acceleration during Walking to Pain and Impact Loading in Adults with Knee Osteoarthritis. Clin. Biomech..

[18] Rogowski I., Creveaux T., Triquigneaux S., Macé P., Gauthier F., Sevrez V. (2015). Tennis Racket Vibrations and Shock Transmission to the Wrist during Forehand Drive. PLoS ONE.

[19] Wei S.-H., Chiang J.-Y., Shiang T.-Y., Chang H.-Y. (2006). Comparison of Shock Transmission and Forearm Electromyography Between Experienced and Recreational Tennis Players During Backhand Strokes. Clin. J. Sport Med..

[20] Nesbit S.M. (2005). A Three Dimensional Kinematic and Kinetic Study of the Golf Swing. J. Sports Sci. Med..

[21] Thain E. (2012). Science and Golf IV.

[22] Evenson K.R., Scherer E., Peter K.M., Cuthbertson C.C., Eckman S. (2022). Historical Development of Accelerometry Measures and Methods for Physical Activity and Sedentary Behavior Research Worldwide: A Scoping Review of Observational Studies of Adults. PLoS ONE.

[23] Pedro B., Cabral S., Veloso A.P. (2021). Concurrent Validity of an Inertial Measurement System in Tennis Forehand Drive. J. Biomech..

[24] Zhang J.-T., Novak A.C., Brouwer B., Li Q. (2013). Concurrent Validation of Xsens MVN Measurement of Lower Limb Joint Angular Kinematics. Physiol. Meas..

[25] Armitage M., Beato M., McErlain-Naylor S.A. (2021). Inter-Unit Reliability of IMU Step Metrics Using IMeasureU Blue Trident Inertial Measurement Units for Running-Based Team Sport Tasks. J. Sports Sci..

[26] Burns A., Greene B.R., McGrath M.J., O’Shea T.J., Kuris B., Ayer S.M., Stroiescu F., Cionca V. (2010). SHIMMER^TM^—A Wireless Sensor Platform for Noninvasive Biomedical Research. IEEE Sens. J..

[27] Mehmood A., Raza A., Nadeem A., Saeed U. (2022). Study of Multi-Classification of Advanced Daily Life Activities on SHIMMER Sensor Dataset. Int. J. Commun. Netw. Inf. Secur..

[28] (2012). JCMG International Vocabulary of Metrology—Basic and General Concepts and Associated Terms (VIM).

[29] Roy R., Paulraj A., Kailath T. (1986). ESPRIT--A Subspace Rotation Approach to Estimation of Parameters of Cisoids in Noise. IEEE Trans. Acoust. Speech Signal Process..

[30] Nijmeijer E.M., Heuvelmans P., Bolt R., Gokeler A., Otten E., Benjaminse A. (2023). Concurrent Validation of the Xsens IMU System of Lower-Body Kinematics in Jump-Landing and Change-of-Direction Tasks. J. Biomech..

[31] Delgado-García G., Vanrenterghem J., Ruiz-Malagón E.J., Molina-García P., Courel-Ibáñez J., Soto-Hermoso V.M. (2021). IMU Gyroscopes Are a Valid Alternative to 3D Optical Motion Capture System for Angular Kinematics Analysis in Tennis. Proc. Inst. Mech. Eng. Part P J. Sports Eng. Technol..

[32] Bosio C., Chadefaux D., Sauret C., Thoreux P. (2024). On-board accelerometers, advancing in-field Biomechanics understanding. Multidiscip. Biomech. J..

